# IMPLEMENTATION BOOSTER: REFLECTION AND ACTION AT THE FRONTLINE

**DOI:** 10.1093/geroni/igae098.0480

**Published:** 2024-12-31

**Authors:** Mary Dolansky, Kevin Little, Lilia Pino, Jennifer Nabong, Anna Bender

**Affiliations:** Case Western Reserve University, Cleveland, Ohio, United States; Informing Ecological Design, LLC, Madison, Wisconsin, United States; CVS Health Minute Clinic, Woonsocket, Rhode Island, United States; CVSHealth Minute Clinic, Woonsocket, Rhode Island, United States; University of Washingotn, Seattle, Washington, United States

## Abstract

Frontline feedback during the implementation of the Age-Friendly Health System (AFHS) 4Ms Framework at MinuteClinic, indicated some providers faced barriers and challenges in delivering 4Ms care. In response, an interdisciplinary team of educators, providers, and other quality improvement experts devised the Reflection and Action strategy, aimed to enhance both competency and confidence among providers in delivering the 4Ms—namely, Mobility, Medications, Mentation, and What Matters Most. Leveraging the well-established “Plan, Do, Study, Act” model for improvement, the team conducted three small-scale experiments to test the Reflection and Action strategy. Each experimental cycle sought to clarify provider expectations, raise awareness of current performance, and catalyze actions for performance improvement during weekly tests of change (inspired by Juran’s Model for Improvement, 1961). Cycle 1 demonstrated a notable 12.5% increase in eligible visits with comprehensive documentation of all 4Ms. Cycle 2 built upon this progress, yielding a further 9.8% increase. Cycle 3 achieved a substantial 27.7% increase in adherence to the 4Ms. Between cycles, the implementation team dynamically adjusted strategies, refined targets, and fine-tuned educational materials and coaching messages to optimize effectiveness. Providers actively participated by completing weekly surveys, providing valuable feedback that informed strategic adjustments. Across all cycles, providers reported heightened confidence in completing the 4Ms assessment, coupled with a reduction in the time required for documentation.

